# Supplementary material for: The patient satisfaction in primary care consultation—Questionnaire (PiC): An instrument to assess the impact of patient-centred communication on patient satisfaction
Source: PLoS One. 2021 Jul 16;16(7):e0254644. doi: 10.1371/journal.pone.0254644 (PMC8284638; doi:10.1371/journal.pone.0254644)
Supplement: S1 File — (PDF) [file pone.0254644.s001.pdf]

EvaSys	Patientenzufriedenheit in der hausärztlichen Konsultation	

Bitte so markieren: ☐ ☒ ☐ ☐ ☐ Bitte verwenden Sie einen Kugelschreiber oder nicht zu starken Filzstift. Dieser Fragebogen wird maschinell erfasst.

Korrektur: ☐ ■ ☐ ☒ ☐ Bitte beachten Sie im Interesse einer optimalen Datenerfassung die links gegebenen Hinweise beim Ausfüllen.

1. Ihre Zufriedenheit im ärztlichen Gespräch

Sehr geehrte Patientin, sehr geehrter Patient,

Ihr Hausarzt nimmt an einer Studie des Forschungsnetzwerkes des Allgemeinmedizinischen Instituts des Universitätsklinikums Erlangen teil. Im Rahmen dieser Studie möchten wir Sie zu Ihrer **Zufriedenheit mit dem ärztlichen Gespräch bei Ihrem heutigen Arztbesuch** befragen.

Unser Ziel ist es, das Verhältnis von Ärzten und ihren Patienten zu erforschen und zu verbessern. Mit Ihren Angaben tragen Sie einen wichtigen Teil dazu bei. Wir bitten Sie daher recht herzlich folgenden Fragebogen ehrlich auszufüllen.
Es können keinerlei Rückschlüsse auf Ihre Person gezogen werden!

Die Ergebnisse der Patientenbefragung werden Ihnen in der teilnehmenden Praxis nach Beendigung der Studie als Aushang zur Verfügung gestellt.

Ihr Allgemeinmedizinisches Institut Erlangen,
im Namen aller Projektbeteiligten des Forschungsnetzwerkes PRO PRICARE

Hinweis:
Versuchen Sie beim Ausfüllen des Fragebogens bitte jede Frage zu beantworten und lassen Sie, wenn möglich, keine aus. Kreuzen Sie bitte je Frage nur eine Antwort an. Bei Unklarheiten wenden Sie Sich bitte gerne an das Praxisteam.

Vielen Dank!

EvaSys	Patientenzufriedenheit in der hausärztlichen Konsultation	

2. Bitte kreuzen Sie an, welche Antwort für Sie <u>nach dem heutigen Gespräch</u> mit Ihrem Hausarzt am ehesten zutrifft.

Modul A

		Stimme voll und ganz zu	Stimme zu	Stimme nicht zu	Stimme überhaupt nicht zu	Weiß nicht
2.1	Hatten Sie das Gefühl, dass Sie mit Ihrem Arzt sprechen konnten?	☐	☐	☐	☐	☐
2.2	Hatten Sie das Gefühl von Ihrem Arzt ernst genommen zu werden?	☐	☐	☐	☐	☐
2.3	Wurden Sie von Ihrem Arzt über verschiedene Behandlungsmöglichkeiten aufgeklärt?	☐	☐	☐	☐	☐
2.4	Wollten Sie über verschiedene Behandlungsmöglichkeiten aufgeklärt werden?	☐	☐	☐	☐	☐
2.5	Wurden Ihnen die Schritte der Behandlung von ihrem Arzt verständlich erklärt?	☐	☐	☐	☐	☐
2.6	Können Sie den Ansatz Ihrer Behandlung nachvollziehen?	☐	☐	☐	☐	☐
2.7	Konnten Sie mit Ihrem Arzt über verschiedene Behandlungsmöglichkeiten diskutieren?	☐	☐	☐	☐	☐
2.8	Sind Sie mit der Entscheidung zu Ihrer weiteren Behandlung zufrieden?	☐	☐	☐	☐	☐
2.9	Wurden Ihre Ideen zu Ihren Beschwerden berücksichtigt?	☐	☐	☐	☐	☐
2.10	Wurden Ihre persönlichen Ängste zu Ihren Beschwerden berücksichtigt?	☐	☐	☐	☐	☐
2.11	Wurden Ihre **Erwartungen an den Umgang** mit Ihren Beschwerden berücksichtigt?	☐	☐	☐	☐	☐

EvaSys	Patientenzufriedenheit in der hausärztlichen Konsultation	

3. Bitte kreuzen Sie an, was Sie Sich generell von einem ärztlichen Gespräch bei Ihrem Hausarzt erwarten.

Modul B

		Stimme voll und ganz zu	Stimme zu	Stimme nicht zu	Stimme überhaupt nicht zu	Weiß nicht
3.1	Ich sollte die Möglichkeit haben mit dem Arzt zu sprechen.	☐	☐	☐	☐	☐
3.2	Mein Standpunkt sollte **mindestens gleich viel wert** sein, wie der des Arztes.	☐	☐	☐	☐	☐
3.3	Der Arzt sollte über alle Behandlungsmöglichkeiten aufklären.	☐	☐	☐	☐	☐
3.4	Der Arzt sollte die Behandlungsinhalte verständlich erklären.	☐	☐	☐	☐	☐
3.5	Ich möchte den Behandlungsweg nachvollziehen können.	☐	☐	☐	☐	☐
3.6	Ich möchte verschiedene Behandlungsmöglichkeiten mit dem Arzt diskutieren können.	☐	☐	☐	☐	☐
3.7	Ich vertraue in der Entscheidungsfindung **voll und ganz** auf die Meinung des Arztes.	☐	☐	☐	☐	☐
3.8	Unabhängig von meinen eigenen Ideen zu meinen Beschwerden, sollte sich mein Arzt sein **eigenes Urteil** bilden.	☐	☐	☐	☐	☐
3.9	Der Arzt sollte meine persönlichen Sorgen zu meinen Beschwerden berücksichtigen.	☐	☐	☐	☐	☐
3.10	Ich möchte meine Erwartungen an den Arzt herantragen.	☐	☐	☐	☐	☐

4. Bitte kreuzen Sie an, was für Sie am ehesten zutrifft.

Modul C

	Sehr zufrieden	Zufrieden	Nicht zufrieden	Überhaupt nicht	Weiß nicht
4.1 Wie zufrieden waren Sie mit dem **heutigen Praxisbesuch** im Allgemeinen?	☐	☐	☐	☐	☐
4.2 Wie zufrieden waren Sie mit dem **heutigen ärztlichen Gespräch** im Allgemeinen?	☐	☐	☐	☐	☐

4.3 Wie lange schätzen Sie die **Dauer des heutigen Arztgespräches** ein ?

☐ Unter 5 Minuten ☐ 5 bis 10 Minuten ☐ > 10 bis 15 Minuten
☐ Über 15 Minuten ☐ Weiß nicht

4.4 Wie viel Zeit sollte Ihrer Meinung nach für ein **Arztgespräch bei allgemeinen Beratungsanlässen** (Bsp.: Rückenschmerzen, Husten, usw.) eingeplant werden?

☐ Unter 5 Minuten ☐ 5 bis 10 Minuten ☐ > 10 bis 15 Minuten
☐ Über 15 Minuten ☐ Weiß nicht

	Zu kurz	Etwas zu kurz	Genau richtig	Etwas zu lang	Zu lang	Weiß nicht
4.5 Wie fanden Sie die **Dauer des heutigen Arztgespräches**?	☐	☐	☐	☐	☐	☐

5. Zusatzfragen - Modul D

5.1 Bitte geben Sie Ihr Geburtsjahr an:

[ ][ ][ ][ ]

5.2 Bitte geben Sie Ihr Geschlecht an:

☐ Weiblich ☐ Männlich
☐ Divers ☐ Keine Angabe

5.3 Bitte geben Sie an, ob Sie privat oder gesetzlich krankenversichert sind:

☐ Privat ☐ Gesetzlich
☐ Keine Angabe

5.4 Sonstige Anmerkungen:

Herzlichen Dank für Ihre Teilnahme!
