# Supplementary material for: The patient satisfaction in primary care consultation—Questionnaire (PiC): An instrument to assess the impact of patient-centred communication on patient satisfaction
Source: PLoS One. 2021 Jul 16;16(7):e0254644. doi: 10.1371/journal.pone.0254644 (PMC8284638; doi:10.1371/journal.pone.0254644)
Supplement: S2 File — (PDF) [file pone.0254644.s002.pdf]

SAMPLE

EvaSys	Patient Satisfaction in the Primary Care Consultation	

PiC

Mark: ☐ ☒ ☐ ☐ ☐ Please use a ballpoint pen or a felt-tip marker. The questionnaire will be captured mechanically.
Correct: ☐ ■ ☐ ☒ ☐ Please pay attention to the indications given on the left in order to ensure optimal data collection.

1. Your satisfaction with the medical consultation

Dear patients,
your general practitioner (GP) is participating in a study conducted by the research network of the Institute of General Practice of the University Hospital Erlangen. Within the scope of the study we would like to register **your satisfaction with the medical consultation based on your appointment today**.

It is our goal to evaluate and improve the relationship between GPs and their patients. Your contribution is a major part of that. We would kindly ask you to fill in the questionnaire honestly. There will be no possibility of tracing the answers back to you!

If you have any questions or a special interest in the study, we will of course be at your disposal.

The results of the patient survey will be sent to the participating practice after the completion of the survey.

Yours sincerely, the Institute of General Practice
in the name of all participating partners of the research network PRO PRICARE

Information: While filling out the questionnaire, please try to answer every question and try to not skip one. Please check only one answer per question. When unsure, please do not hesitate to turn to the practice team for help.

Thank you very much!

SAMPLE

SAMPLE

2. Please indicate which answer is most applicable after today's consultation with your GP.

Module A

		Fully agree	Agree	Disagree	Fully disagree	Do not know
2.1	Did you feel as though you were able to talk to your GP?	☐	☐	☐	☐	☐
2.2	Did you feel as though you were taken seriously by your GP?	☐	☐	☐	☐	☐
2.3	Did your GP explain various treatment options?	☐	☐	☐	☐	☐
2.4	Did you want to know more about various treatment options?	☐	☐	☐	☐	☐
2.5	Did your GP explain the steps of the treatment in a comprehensive manner?	☐	☐	☐	☐	☐
2.6	Were you able to understand the treatment approach?	☐	☐	☐	☐	☐
2.7	Were you able to discuss different treatment options with your GP?	☐	☐	☐	☐	☐
2.8	Are you satisfied with the decision concerning your further treatment?	☐	☐	☐	☐	☐
2.9	Were your ideas concerning your symptoms considered?	☐	☐	☐	☐	☐
2.10	Were your personal fears concerning your symptoms considered?	☐	☐	☐	☐	☐
2.11	Were your **expectations concerning the handling** of your symptoms considered?	☐	☐	☐	☐	☐

SAMPLE

SAMPLE

3. Please indicate what you generally expect from the consultation with your GP.

Module B

		Fully agree	Agree	Disagree	Fully disagree	Do not know
3.1	I should have the opportunity to talk to the GP.	☐	☐	☐	☐	☐
3.2	My own point of view should be **worth at least as much** as that of the GP.	☐	☐	☐	☐	☐
3.3	The GP should explain all treatment options.	☐	☐	☐	☐	☐
3.4	The GP should explain all treatments comprehensively.	☐	☐	☐	☐	☐
3.5	I want to able to understand the treatment procedure.	☐	☐	☐	☐	☐
3.6	I want to be able to discuss various treatment options with the GP.	☐	☐	☐	☐	☐
3.7	I trust the decision making and the opinion of my GP **fully**.	☐	☐	☐	☐	☐
3.8	My GP should form their **own opinion**, regardless of my personal ideas concering my symptoms.	☐	☐	☐	☐	☐
3.9	The GP should consider my personal fears regarding my symptoms.	☐	☐	☐	☐	☐
3.10	I want to be able to present my expectations to my GP.	☐	☐	☐	☐	☐

SAMPLE

SAMPLE

4. Please indicate which options is the most applicable to you.

Module C

	Very satisfied	Satisfied	Dissatisfied	Very dissatisfied	Do not know
4.1 How satisfied were you with **today's practice visit** in general?	☐	☐	☐	☐	☐
4.2 How satisfied were you with **today's medical consultation** in general?	☐	☐	☐	☐	☐

4.3 How long do you estimate the **duration of the consultation**?

☐ Under 5 minutes ☐ 5 to 10 minutes ☐ > 10 to 15 minutes
☐ Over 15 minutes ☐ Do not know

4.4 How long do you think the duration of the consultation should be for **general consultation issues** (e.g. back pain, cough etc.)?

☐ Under 5 minutes ☐ 5 to 10 minutes ☐ > 10 to 15 minutes
☐ Over 15 minutes ☐ Do not know

	Too short	Slightly too short	Adequate	Slightly too long	Too long	Do not know
4.5 How did you find the **duration of today's consultation**?	☐	☐	☐	☐	☐	☐

5. Additional questions - Module D

5.1 Please indicate your year of birth:

[ ][ ][ ][ ]

5.2 Please indicate your sex:

☐ Female ☐ Male
☐ Divers ☐ No indication

5.3 Please indicate whether you have private or statutory health insurance:

☐ Private ☐ Statutory
☐ Keine Angabe

5.4 Further remarks:

[ ]

Thank you very much for your participation!

SAMPLE
